# Perspectives on Reducing Barriers to the Adoption of Digital and Computational Pathology Technology by Clinical Labs

**DOI:** 10.3390/diagnostics15070794

**Published:** 2025-03-21

**Authors:** Jeffrey L. Bessen, Melissa Alexander, Olivia Foroughi, Roderick Brathwaite, Emre Baser, Liam C. Lee, Omar Perez, Gary Gustavsen

**Affiliations:** 1 Health Advances LLC, Newton, MA 02466, USA; 2 AstraZeneca, Gaithersburg, MD 20878, USA

**Keywords:** digital and computational pathology, anatomic pathology, pathologists, lab directors, AI-guided image analysis, whole-slide imaging, implementation, adoption, survey

## Abstract

**Background/Objectives:** Digital and computational pathology (DP/CP) tools have the potential to improve the efficiency and accuracy of the anatomic pathology workflow; however, current adoption among US hospital and reference labs remains low. **Methods:** To better understand the current utilization of DP/CP technology and barriers to widespread adoption, we conducted a survey among 63 anatomic pathologists and lab directors within the US health system. **Results:** The survey results indicated that current use cases for DP/CP involve streamlining traditional manual pathology and that labs would have substantial difficulty providing AI-guided image analysis if it were required by physicians today. Among potential catalysts for the broader adoption of DP/CP, pathologists identified clinical guidelines as a key resource for anatomic pathology, whose endorsement of DP/CP would be highly impactful for reducing current barriers. **Conclusions:** Expanded access to DP/CP may ultimately benefit all major stakeholders—patients, physicians, clinical laboratory professionals, care settings, and payers—and will therefore require collaboration across these groups.

## 1. Introduction

Digital and computational pathology (DP/CP) tools are on the verge of becoming a necessity for anatomic pathology (AP) labs [1,2] to improve efficiency and ensure patient access to ground-breaking personalized medicines. DP/CP encompasses a range of hardware and software that enables labs to digitize glass slides, archive scanned slides, annotate digital images, and perform algorithmic analysis. DP/CP has the potential to address many current challenges with manual anatomic pathology: a shortage of pathologists [3], growing volume and complexity of AP cases [4], inefficiencies storing and retrieving glass slides [5], and significant variability in biomarker scoring between pathologists [6,7]. Additionally, AI-guided image analysis is being studied on a clinical trial sample as a potential companion diagnostic [8,9] and could soon become clinically actionable and necessary for determining a patient’s eligibility for next-gen targeted therapies.

However, evidence suggests that current adoption of DP/CP workflows among labs and particularly for primary diagnosis is low [2,10], driven by high implementation costs, lack of reimbursement, a need for dedicated personnel and space, as well as digital storage, IT integration requirements, and pathologist hesitancy. It can be expensive to establish and maintain DP/CP capabilities in the lab, with high upfront costs to purchase capital equipment and ongoing fees for the software necessary to view and store images [11]. Reimbursement is a major challenge—while there are CPT codes unique to DP/CP [12], they are not preferentially reimbursed, compared to manual pathology codes, or are too new to have fees associated with them [13]. DP/CP requires adaptation by pathologists [14], dedicating full time employees (FTEs) and workspace to digitize and analyze slides [2], and costly digital storage that must be compliant with all regulations governing storing healthcare data [15]. IT integration requires cross-disciplinary coordination to integrate DP/CP tools with Laboratory Information Management Systems (LIMS) and the Electronic Health Record (EHR) [16]. Finally, there is hesitancy among pathologists [1], many of whom feel more comfortable with the manual pathology workflows they trained with.

To better understand the current drivers and barriers to the adoption of DP/CP in the US and potential catalysts for increasing uptake in the near future, we conducted a quantitative survey among anatomic pathologists practicing at a range of institutions. The study revealed three overarching findings. First, DP/CP is mainly used today to streamline traditional manual pathology tasks, such as sharing images at the tumor board and archiving patient slides, while few labs are routinely performing CP. Second, pathologists—even those with hands-on experience with CP—would have substantial difficulty providing AI-guided algorithmic analysis if it were requested by physicians today, driven by perceptions around the nascency of the technology and paucity of clinical evidence. Third, pathologists believe clinical guidelines by bodies such as CAP and NCCN may play a substantial role in expanding the awareness and credibility of DP/CP technologies, driving adoption within hospital labs and facilitating reimbursement analyses for payers.

## 2. Materials and Methods

Survey development was informed by preliminary 60 min qualitative phone interviews with a small subset of anatomic pathologists (*n* = 5)—serving in staff pathologist and/or lab administrator roles—focusing on the current adoption of DP/CP technology and the barriers and potential catalysts for broader uptake.

A survey of a broader group of US pathologists (*n* = 63) was conducted in July 2024. To ensure high-quality market research, the survey respondents were recruited to take an online survey by a market research insight collection company (M3 Global Research) in a double-blinded fashion so that the identity of the respondent was not revealed to us (the sponsor), and the sponsor of the study was not revealed to the respondent in compliance with industry standards.

To participate, the respondents were required to be employed as a laboratory director, laboratory manager/supervisor, or staff pathologist at a clinical laboratory; hold responsibilities including developing and supervising laboratory workflows; oversee histopathology and/or cytopathology; have practiced as an anatomic pathologist; and have some familiarity with digital pathology. If participants met the screening criteria, participants proceeded to complete a 20 min questionnaire (Data Supplement in Supplementary Materials) and received a USD 60 honorarium for completing the survey. Respondent demographics are displayed in Table 1.

After fielding the survey, additional 60 min qualitative phone interviews with selected survey respondents were conducted (*n* = 6) in a double-blinded fashion in order to obtain further context behind the respondent’s answers to the survey questions and to test the hypothesis stemming from the analysis of the survey data.

## 3. Results

### 3.1. Current Trends in Anatomic Pathology

**Figure 1:**
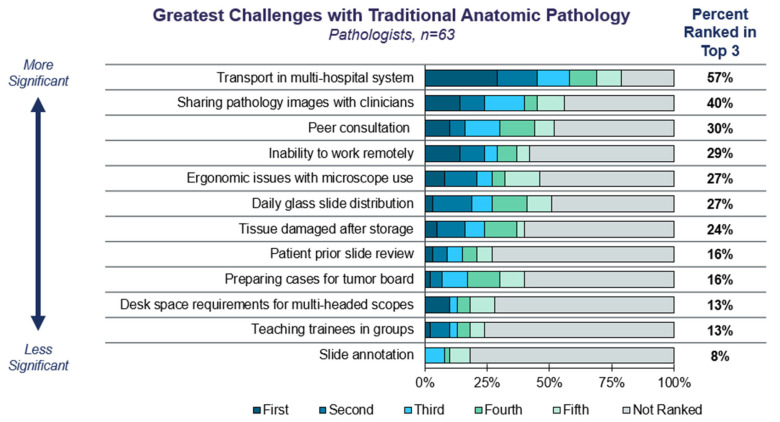
**Challenges with traditional anatomic pathology**. Survey respondents were asked to select and rank the challenges with traditional manual pathology based on a provided list of options.

We began the survey by exploring current challenges with manual anatomic pathology. Pathologists reported that top pain points with traditional manual pathology today are related to the logistical difficulties of sharing slides between pathologists or challenges of accessing images remotely (Figure 1). Sharing slides is particularly important within multi-hospital systems, where pathologists may reside at only one hospital within the network or work at multiple hospitals within the system. Slide sharing also facilitates clinical decision-making—such as reviewing images at a multidisciplinary tumor board—and consultation with specialized pathologists practicing elsewhere (i.e., ‘telepathology’).

We then defined the different elements of DP/CP (Supplementary Figure S1) and probed the extent of DP/CP utilization among survey respondents. We found surprisingly high self-reported levels of DP/CP adoption, especially for more advanced-use cases, such as image viewing and annotation and AI-guided image analysis (Supplementary Table S1). There are multiple potential reasons that this could be: the survey explicitly screened for pathologists with some DP/CP experience; self-selection bias may have occurred if the adopting pathologists were more likely to complete the survey than non-adopters; or market research bias. We also suspected, and later confirmed in interviews with select survey respondents, that pathologists reported adopting elements of DP/CP, even if the elements were not in routine clinical use, e.g., usage for research or validation purposes.

**Figure 2:**
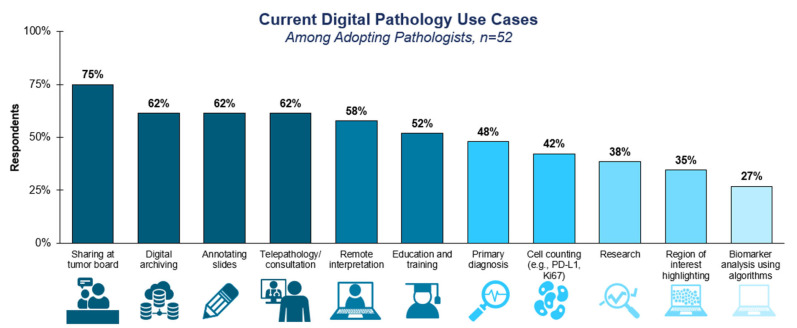
**Current digital pathology use cases**. Survey respondents were asked for which of the listed use cases they were using digital pathology for today, selecting all options that applied. The question was only asked of respondents who indicated in a previous survey question that they had adopted one or more elements of digital pathology in their lab. Shading of the bar graph was used to indicate deciles.

Currently, among adopting labs, digital pathology is most commonly used to streamline traditional manual pathology tasks, such as tumor board and archiving slides, while fewer labs use nascent techniques, such as automated scoring (Figure 2). A total of 75% of surveyed labs reported using digitized slide images at tumor boards, while 62% used digital archiving, slide annotation, and/or telepathology.

### 3.2. Adoption Considerations for Computational Pathology

**Figure 3:**
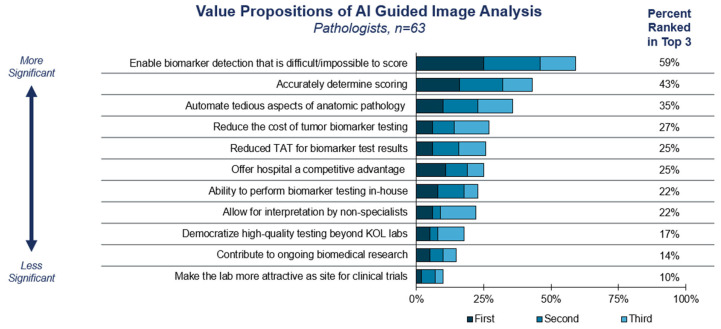
**Value propositions of AI-guided image analysis**. Survey respondents were asked to select and rank the value propositions of AI-guided image analysis based on a provided list of options.

We next sought to understand the drivers and barriers to the adoption of AI-guided image analysis (also referred to as computational pathology or CP). The top value proposition for AI-guided analysis is to enable biomarker detection that is difficult or impossible to score manually, such as PD-L1 (Figure 3). Pathologists also believed it may improve the accuracy of scoring and automate tedious aspects of AP, such as mitotic counting. In short, the strongest appeal of CP is its potential to match or exceed the performance of traditional manual pathology.

**Figure 4:**
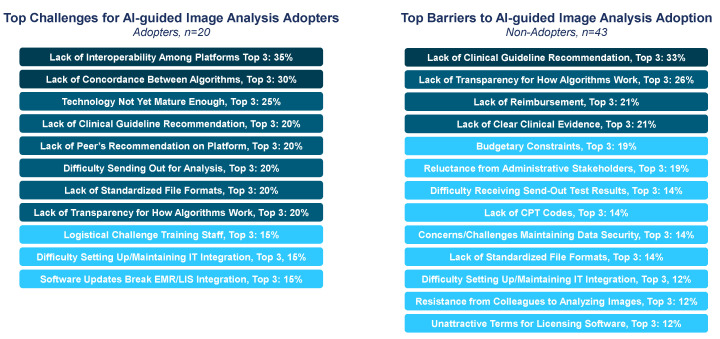
**Challenges associated with computational pathology**. Survey respondents were asked to select and rank the top challenges (among adopters) or barriers to adoption (among non-adopters) associated with AI-guided image analysis, based on a provided list of options. Shading of boxes is used to indicate deciles. Answers chosen by <10% of respondents are not shown.

However, pathologists reported significant challenges with implementing CP, even among labs that had first-hand experience with the algorithms. Among labs that had not adopted CP, the top challenges for adoption included a lack of guideline recommendations, followed by lack of transparency for how the algorithms worked, reimbursement uncertainty, and inadequate clinical evidence (Figure 4). For labs experienced with CP—via use in clinical practice or for research/validation purposes—the top challenges included a lack of interoperability among DP/CP platforms, as well as a lack of concordance between similar algorithms.

We then sought a deeper understanding of the implementation of AI-guided image analysis by adopting labs. Among adopters, there were several markers (HER2, PD-L1, and KI-67) that were scored via algorithms (Figure S2A). Validation typically occurred via a combination of internal and published validation studies and required an average of 5 months and 4.4 full-time equivalents (FTEs; Figure S2B–D), representing a significant investment of time and resources by the lab.

**Figure 5:**
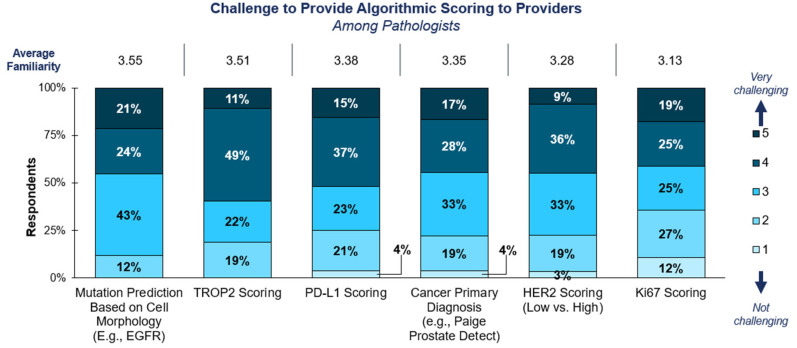
**Challenge of providing AI algorithmic analysis to providers**. Survey respondents were asked to rate the difficulty of providing algorithmic scoring on a solid tumor specimen if requested by a provider in the near future. Respondents were only asked about markers with which they had indicated familiarity in a prior survey question.

According to the surveyed pathologists, the vast majority of labs would struggle to provide AI slide analysis today (Figure 5). Across all solid tumor markers, more than 75% of responses rated performing CP as a 3 or higher, indicating it would be somewhat-to-very challenging to perform such an analysis on behalf of providers. Both adopters and non-adopters perceived the technology and evidence for CP as not yet sufficiently mature for clinical use (Supplementary Figure S3). Other challenges to the near-term adoption of CP included budgetary constraints preventing in-house adoption, lack of reimbursement for CP, and difficulties coordinating the results between the originating and performing labs.

### 3.3. Role for Guidelines in Promoting DP/CP Adoption

In our investigation, clinical guidelines emerged as a critical resource for pathologists staying up to date on advances in pathology, as well as a potential catalyst for DP/CP adoption. Specifically, pathologist-facing guidelines (CAP and AMP), as well oncology guidelines (NCCN and ASCO), were seen as the most influential resources by pathologists (Supplementary Figure S4). Beyond guidelines, other key resources included colleagues (such as at the tumor board or conferences) and clinical literature.

**Figure 6:**
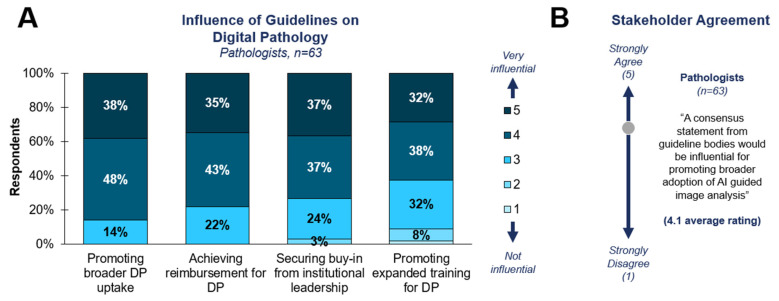
**Influence of guidelines on digital pathology**. (**A**) Survey respondents were asked to rate the influence of guideline body recommendations for digital pathology for each factor. (**B**) Survey respondents were asked to rate their level of agreement with the provided statement.

Given the influence of guidelines, lab directors believed that guideline inclusion for digital pathology would have a meaningful impact on future adoption, reimbursement, and training. For pathologists, 75+% saw guidelines as influential (indicated by selecting 3 or higher) for promoting broader uptake, reimbursement, buy-in, and training for DP (Figure 6A). Additionally, 81% of survey respondents agreed that a consensus statement from guideline bodies would be influential for promoting the broader adoption of AI-guided image analysis (Figure 6B).

Over the next 3–5 years, pathologists expect to increase their use of DP/CP across each of the four elements (Supplementary Figure S5). Pathologists anticipate that biomarker analysis using algorithms will become a more common use case in the future (Supplementary Figure S6). Guideline incorporation, as well as reimbursement and billing support, are seen as the top drivers for reducing barriers to incorporating AI image analysis in the future (Supplementary Figure S7).

## 4. Discussion

This large-scale survey-based study of US pathologists and laboratory directors establishes that significant challenges remain to ensuring that patients have access to DP/CP technologies. Despite the hurdles to DP/CP adoption, interest is steadily increasing over time [17,18], as is market size, which is projected in one report to reach USD 2,082.7 million by 2032 [19]. This is in part due to the increasing recognition of value attributed to the most common use cases that the current and prior publications have highlighted, such as slide sharing, archiving, and telepathology (Figure 1) [20,21]. Other factors are also contributing to the increasing interest in DP/CP. For example, the use of biomarkers to guide therapy decisions is growing in number and complexity and is occurring on the backdrop of increased specimen volumes, laboratory financial strains, and a shrinking workforce of pathologists. It is therefore not unexpected that the current study reveals that biomarker scoring is ranked at the top for value for AI-guided image analysis adoption (Figure 2). Among its many attributes, DP/CP has the potential to both automate mundane tasks and also provide standardized biomarker scoring assistance to reduce variabilities within any given lab, between labs, and between pathologists.

DP/CP guideline and practice recommendations from primary governing bodies (e.g., CAP) have long been an essential resource for the standardization of pathological staging and reporting and now serve a similar function for biomarker evaluation (Supplementary Figure S5). There are a growing number of commercially available and in-house algorithms developed to address both efficiency and variability in diagnostics, as well as biomarker assessment. The current study confirms that pathologists have reservations about incorporating AI into their diagnostic workflow and will be seeking guideline inclusion as a prerequisite for DP/CP adoption (Supplementary Figure S4, Figure 3). Important to note are the signs of progress. Guidelines for validating whole-slide images have been published, as well as guidelines for quantitative image analyses for certain markers [22,23]. Add-on codes for digital pathology have been created, and the list was expanded in 2024, although with little if any reimbursement success to date [12]. Most recently, recommendations for machine learning performance evaluation have been published as a concept paper from CAP [24]. Actions taken by guideline bodies have the potential to directly and indirectly address several of the main challenges labs face in incorporating AI-guided algorithms into practice. Endorsement of CP by guidelines can draw attention to the current and growing body of evidence supporting its use in routine clinical practice [8]. Previous studies have shown that oncology clinical guideline adherence can result in improved outcomes [25], and guideline endorsement and adoption of DP/CP technologies by labs may have a similar positive impact for patients.

The business case for DP/CP adoption is complex due to the unique pressures in any given laboratory setting, such as geography, case volume and diversity, number of practice sites, and myriad other variables [26]. Numerous guides to digital pathology adoption have been published, as have return on investment (ROI) calculators for laboratories [18,27,28]. The most recent ROI tool created in association with the Digital Pathology Association (DPA) is the first publicly available calculator that is exhaustive, customizable, and dynamic, with the ability to adapt to possible changes in reimbursements for current and new technologies [29]. Among the new technologies could be patient selection for therapies based solely on DP/CP algorithms. However, opportunities abound to utilize DP/CP for additional revolutionary precision medicine applications, and the flexibility to incorporate these novel modalities into a laboratory’s budget forecast will be of great value [30,31]. It is important to recognize when evaluating the business case for adopting DP/CP that labs may not need to adopt every element of the workflow to unlock algorithmic scoring capabilities. In the current study, numerous pathologists reported the adoption of image acquisition but not image storage due to cost and privacy issues (Supplementary Table S1). Given this finding, we hypothesize that the only prerequisites for adopting CP may be having access to a slide scanner and an established workflow for digitizing slides, without requiring several of the DP/CP technologies conventionally considered foundational (Supplementary Figure S2). With regard to DP specifically, although reimbursement rates are poor and currently less than manual pathology, there is optimism that with evolving data showing utility and value, success in reimbursement will follow.

Beyond the financial complexities, if an institution is contemplating adopting DP/CP, it is also imperative to recognize the regulatory challenges and considerations that come with it. For example, the Health Insurance and Accountability Act of 1996 also applies to the use of AI and requires the same standards as traditional patient health information. Further complicating the situation is the explosion of DP/AI technologies on many different platforms, which brings an increased likelihood of working with third parties. In response to this risk, safeguards such as business associate agreements will need to guide the use of patient information in this setting. Finally, AI that is used for clinical care (such as an AI biomarker algorithm) falls under the FDA definition of software as a medical device (SaMD), with each device placed into one of three classifications based on risk to patients and regulated accordingly. The complex regulatory processes surrounding SaMD, including issues such as software updates and enhancements, are beyond the scope of this manuscript, and detailed analyses are available elsewhere, but an understanding that the regulatory landscape is constantly evolving in response to new opportunities in AI is critical when considering DP/CP adoption [32].

Ultimately, making progress on DP/CP access will require collaboration across all healthcare stakeholders. Pathologists and lab directors will be responsible for implementing digital and computational pathology workflows in their labs. Prescribers must recognize the need for digital pathology in routine clinical practice, order appropriate tests, and integrate the results into clinical algorithms. Policymakers, regulators, and payers must establish appropriate metrics for achieving approval and coverage for advances in DP/CP, including revisiting whether existing frameworks are appropriate for evaluating software tools. Additionally, pharma and diagnostics sponsors—both individually and via consortia—must generate clinical evidence for the necessity and actionability of DP and establish standards for the interoperability of DP/CP components.

## 5. Conclusions

The field of DP/CP is evolving rapidly due to compelling use cases in efficiency, diagnostic support, and advancement of precision medicine, as demonstrated in this large-scale survey-based study. However, work remains to move the field further into mainstream widespread use. The findings in this manuscript provide a status check for where the landscape is on adoption and the important challenges and support needs that remain. Findings demonstrate an anticipation of growing adoption of these technologies but also highlight that a better understanding of the technology and further support from highly impactful guideline-issuing bodies will be crucial to move forward. Continued input and motivation to adapt by the pathology community will be critical in these efforts.

## Figures and Tables

**Table 1:** **Demographics of survey respondents**. Stakeholders for this study included anatomic pathologists and lab directors. All respondents were 21 years of age or older at the time of the survey. Ethical review for this study was completed by the Advarra CIRBI Platform using the Department of Health and Human Services regulations found at 45 CFR 46.104(d)(2); the IRB determined that the research project is exempt from IRB oversight.

Survey Respondent Demographics		
Pathologists and Lab Directors (*n* = 63)		
		Respondents
Title/Role	Laboratory director	67%
	Laboratory manager/supervisor	3%
	Staff pathologist	30%
Geographic Region	West	33%
	Midwest	16%
	South	27%
	Northeast	24%
Lab Setting	Independent reference lab	6%
	Academic hospital	37%
	Community hospital	46%
	Academic-affiliated community hospital	11%
Current Use of Digital Pathology (DP)	Non-user but familiar with DP	14%
	User of DP for educational purposes only	17%
	User of DP for primary diagnosis only	11%
	User of DP for multiple purposes	57%

## Data Availability

The data underlying this article will be shared upon reasonable request to the corresponding author.

## Supplementary Materials

The following supporting information can be downloaded at: https://www.mdpi.com/article/10.3390/diagnostics15070794/s1, Figure S1: Elements of Digital Pathology; Figure S2: Implementation of AI Guided Image Analysis; Figure S3: Reasons for Challenge with Providing AI Algorithms; Figure S4: Key Resources for Anatomic Pathology; Figure S5: Future Use of Digital Pathology; Figure S6: Current and Future Digital Pathology Use Cases; Figure S7: Keys to Reducing Barriers to AI Image Analysis; Table S1: Self-Reported Adoption of DCP Among Survey Respondents. Supplementary Methods: Pathologist Survey Questionnaire.
