# Supplementary material for: Perspectives on Reducing Barriers to the Adoption of Digital and Computational Pathology Technology by Clinical Labs
Source: Diagnostics (Basel). 2025 Mar 21;15(7):794. doi: 10.3390/diagnostics15070794 (PMC11988507; doi:10.3390/diagnostics15070794)
Supplement: Supplementary file 1 [file diagnostics-15-00794-s001.zip › diagnostics-3505119-supplementary-figures.pdf]

Supplementary Results:

Figure S1

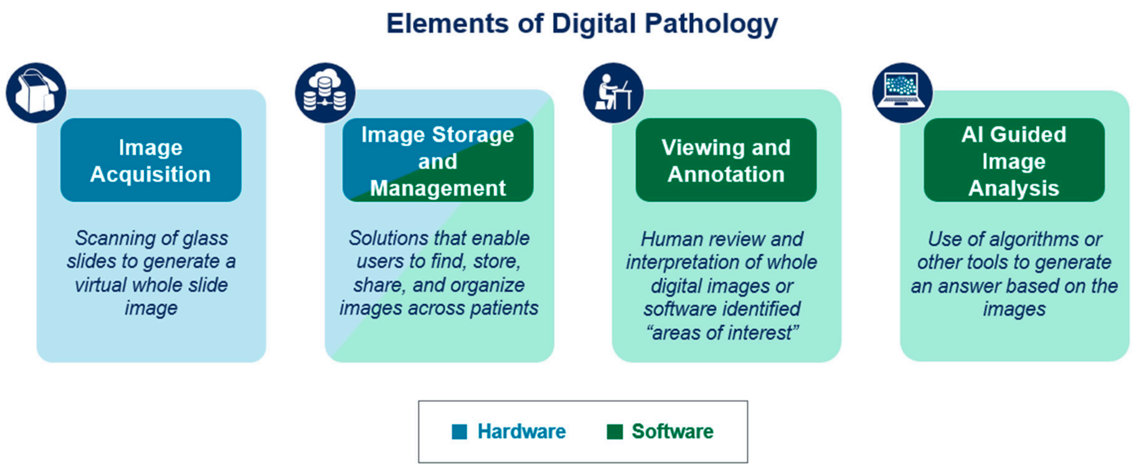

Figure S1: Elements of Digital Pathology. Survey respondents were presented with definitions of each of the key elements of digital pathology.

Table S1: Self-Reported Adoption of DCP Among Survey Respondents. Surveyed pathologists were asked to select which of the elements of digital pathology had been adopted by their lab, selecting all that applied. According to post-survey interviews with respondents, some pathologists considered an element to be 'adopted' by their lab if it had been used for research or validation purposes, whether or not it was in routine clinical use.

**Element**		**Respondents**
Image Acquisition	Adopted	57%
	Not Adopted	43%
Image Storage and Management	Adopted	71%
	Not Adopted	29%
Image Viewing and Annotation	Adopted	65%
	Not Adopted	35%
AI Guided Image Analysis	Adopted	32%
	Not Adopted	68%
None of the above	--	17%

Figure S2

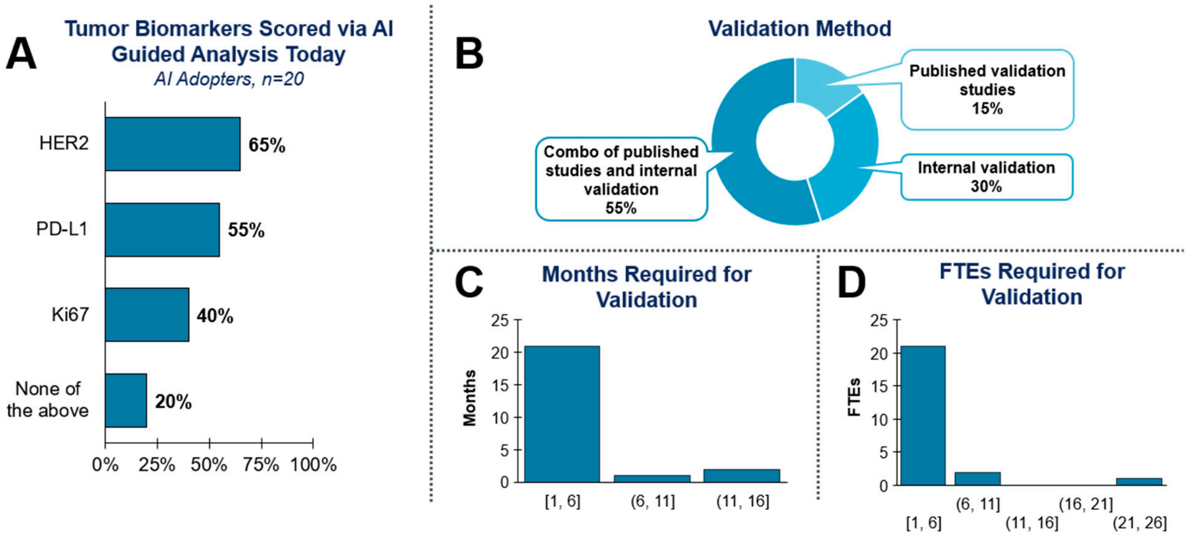

Figure S2: Implementation of AI Guided Image Analysis. A) Survey respondents who previously indicated they had adopted AI guided image analysis were asked which solid tumor markers they had scored algorithmically. Respondents were not prompted to limit their answer to just assays that had been run for routine clinical use. **B-D)** Pathologists were asked how labs validated AI guided image analysis, as well as how many months and full-time equivalents (FTEs) had been required for validation.

Figure S3

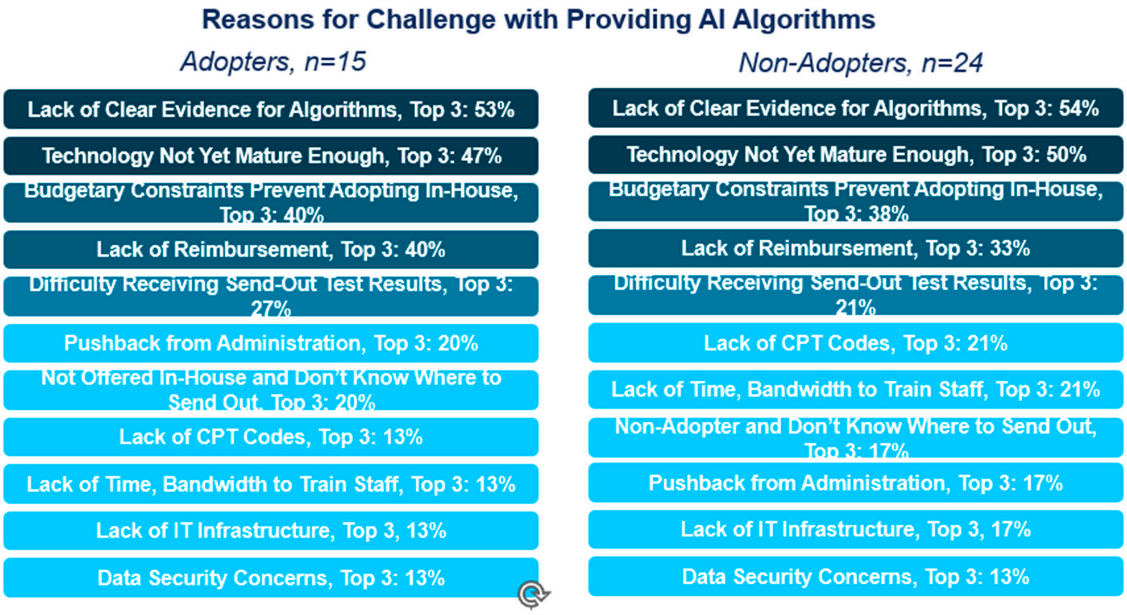

Figure S3: Reasons for Challenge with Providing AI Algorithms. Survey respondents were asked to select and rank the top challenges with providing AI-guided image analysis, based on a provided list of options. Shading of boxes is used to indicate deciles. Questions only shown to respondents who rated the challenge with providing AI algorithms a score of 3 or higher. Answers chosen by <10% of respondents not shown.

Figure S4

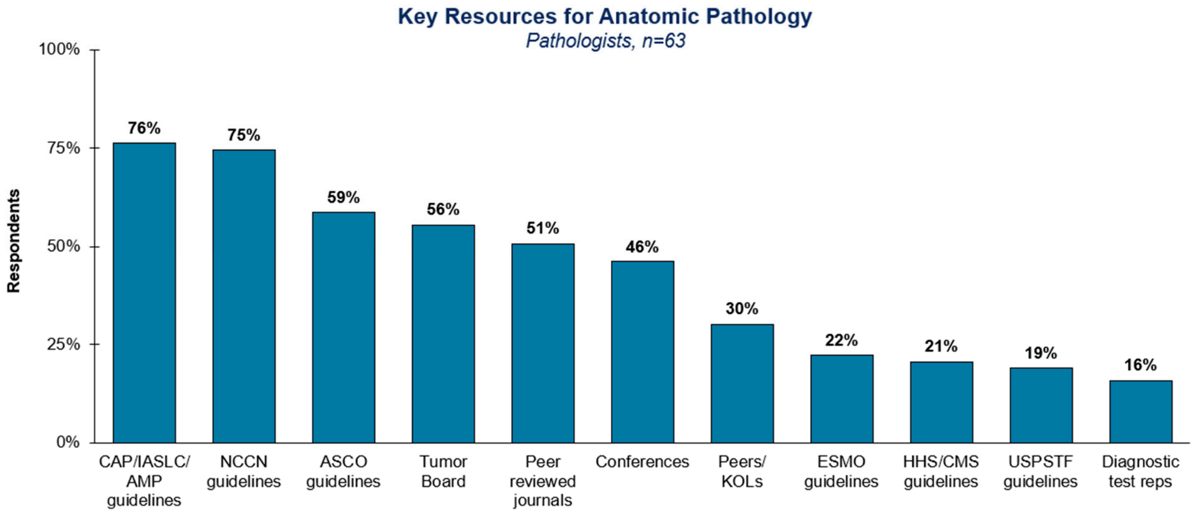

Figure S4: Key Resources for Anatomic Pathology. Survey respondents were asked to choose which of the listed guidelines or resources they use to determine protocols for anatomic pathology, selecting all that apply. Answers chosen by <15% of respondents not shown.

Figure S5

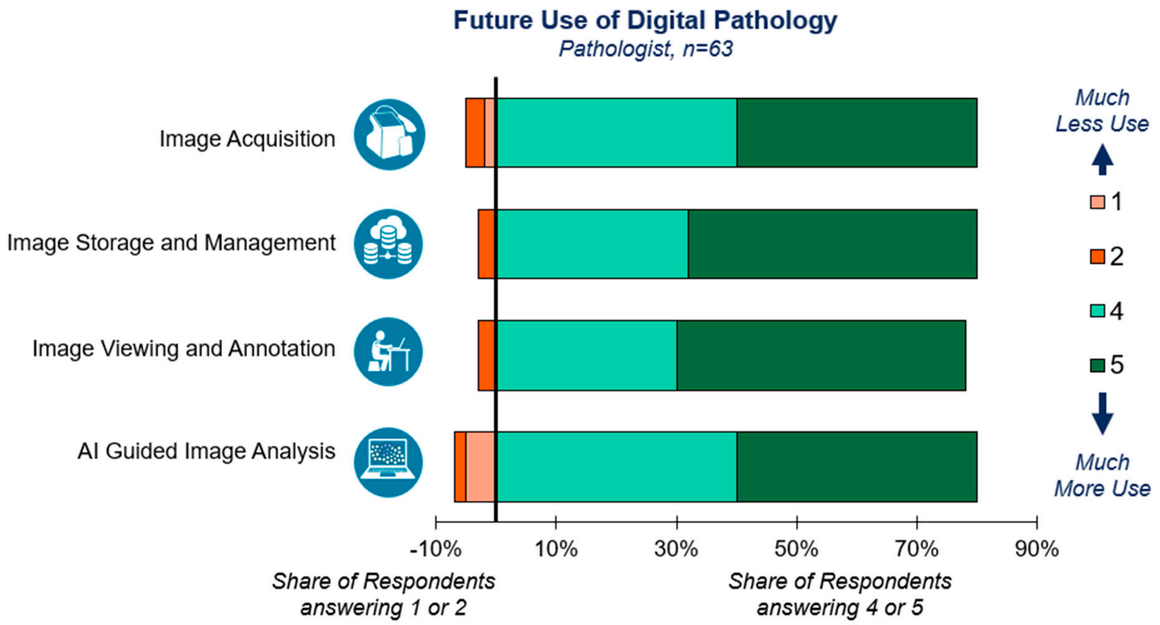

Figure S5: Future Use of Digital Pathology. Survey respondents were asked to indicate their expected future use of digital pathology at their institution over the next 3-5 years.

Figure S6

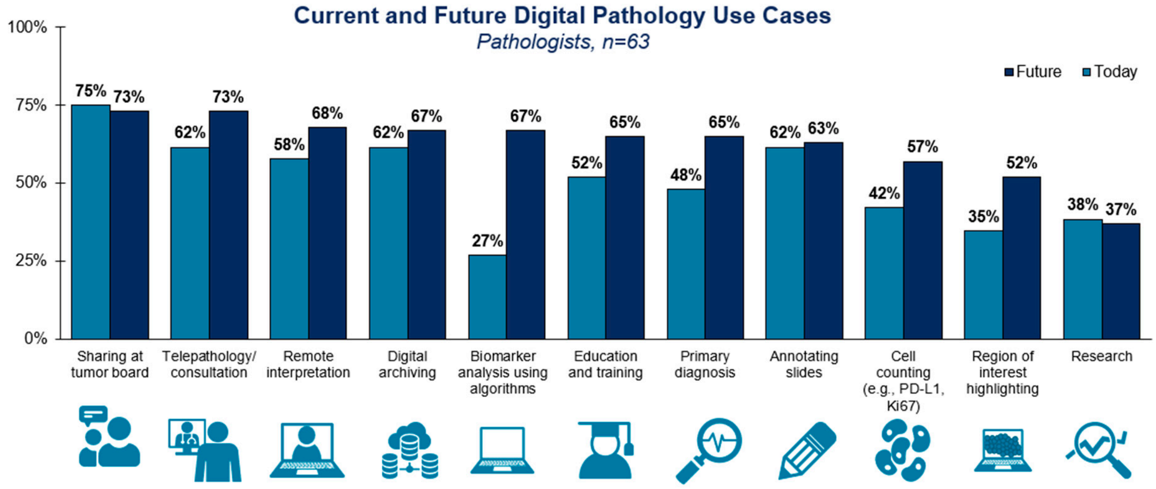

Figure S6: Current and Future Digital Pathology Use Cases. Respondents were asked to select current use cases for digital pathology in their labs (i.e., Figure 1) as well as anticipated use cases in the future.

Figure S7

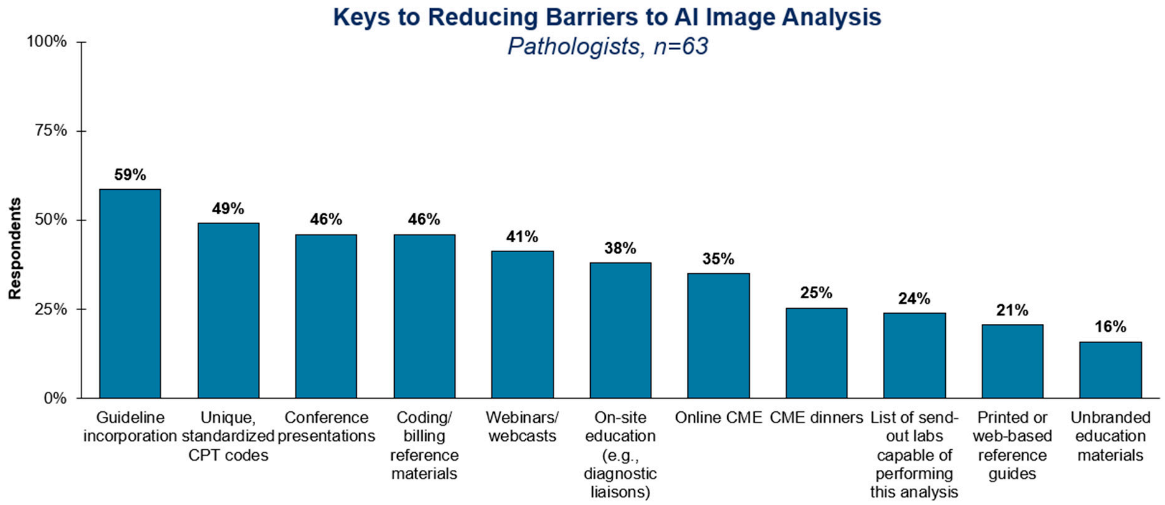

Figure S7: Keys to Reducing Barriers to AI Image Analysis. Survey respondents were asked to choose which of the factors on the provided list would be most impactful for reducing barriers to adoption of AI guided image analysis, selecting all that apply.
