# Supplementary material for: Perspectives on Reducing Barriers to the Adoption of Digital and Computational Pathology Technology by Clinical Labs
Source: Diagnostics (Basel). 2025 Mar 21;15(7):794. doi: 10.3390/diagnostics15070794 (PMC11988507; doi:10.3390/diagnostics15070794)
Supplement: Supplementary file 1 [file diagnostics-15-00794-s001.zip › diagnostics-3505119-supplementary-questionnaire.pdf]

Supplementary Methods: Pathologist Survey Questionnaire

Screener Questions

1. How would you classify your primary laboratory? *Please select only one.*
   a. Research laboratory **[Terminate from Survey if selected]**
   b. Clinical laboratory
   c. Other (please specify): **[Terminate from Survey if selected]**
2. Which of the following most closely matches your title or role?
   a. Laboratory director
   b. Laboratory manager/supervisor
   c. Staff pathologist
   d. Laboratory tech, lead tech, or medical tech **[Terminate from Survey if selected]**
3. How would you classify the institution where your laboratory is located?
   a. Independent reference laboratory
   b. Academic hospital/academic medical center/university
   c. Community hospital
   d. Academic-affiliated community hospital lab
   e. Public health laboratory **[Terminate from Survey if selected]**
   f. Collection center/blood bank **[Terminate from Survey if selected]**
   g. Government hospital or facility **[Terminate from Survey if selected]**
   h. Other (please specify): **[Terminate from Survey if selected]**
4. Which of the following activities are you personally involved in or responsible for managing? *Please select all that apply.*
   a. Supply inventory management
   b. Quality control/quality assurance/compliance
   c. Assay or instrument selection and purchasing
   d. Developing/supervising laboratory workflows **[Terminate from Survey if <u>not</u> selected]**
   e. Reviewing and releasing test results
   f. I do not participate in any of these activities **[Terminate from Survey if selected]**
5. Which of the following activities are you personally involved in or responsible for managing? *Please select all that apply.*
   a. Histopathology
   b. Cytopathology **[Terminate from Survey if A *and* B <u>not</u> selected]**
   c. Clinical chemistry
   d. Immunoassay
   e. Coagulation
   f. Hematology
   g. Urinalysis
   h. Molecular / nucleic acid testing
   i. Microbiology
   j. Other (please specify):
   k. None of the above **[Terminate from Survey if selected]**
6. Do you currently practice, or have you previously practiced, as an anatomic pathologist?
   a. Yes
   b. No **[Terminate from Survey if selected]**
7. In which state is your practice located? **[Terminate from Survey if ME, VT, WV selected from drop-down menu]**
8. [*If practicing in Georgia*] Are you currently employed by the state of Georgia? *Please select only one.*
   a. Yes
   b. No **[Terminate from Survey if selected]**

9. How does your lab use digital pathology today? *Please understand digital pathology to be management and analysis of data derived from digitized anatomic pathology specimen slides.*

a. I am not familiar with digital pathology **[Terminate from Survey if selected]**
b. We do not use digital pathology, but I am familiar with it
c. Our lab uses a scanner for educational purposes, but does not otherwise use it for clinical diagnosis
d. Our lab uses digital pathology for primary diagnosis only
e. Our lab uses multiple types of digital pathology (e.g., telepathology, AI-guided image analysis)

Congratulations! You have qualified for our survey. This survey will take approximately 20 minutes to complete. You must complete the entire survey to qualify for the honorarium.

We are conducting this survey to support development of a publication to raise awareness about digital pathology. In order to successfully carry out this mission, we must have a deep understanding of current adoption trends and barriers to accessing digital pathology in US health systems.

This survey will evaluate practices in and barriers to digital pathology across US health systems. We implore respondents to accurately represent the health systems at which they are employed. We understand that you may wish to confer with others in your health system. You will be able to leave and return to this link to complete the survey at any time.

The possible risks of the study are minimal. Furthermore, your participation is strictly voluntary, and you may withdraw your participation at any time without penalty.

The results of this survey will only be shared on an aggregated, anonymous basis. Your responses will be treated as confidential and will not be shared in any format that could be directly linked back to you or your practice.

By clicking NEXT you are verifying that you have read the explanation of the study, you are over the age of 21, and that you agree to participate. You also understand that your participation in this study is strictly voluntary.

Thank you for your participation.

1. Which of the following guidelines or resources do you use to determine protocols for anatomic pathology? *Please select all that apply.*
   a. ESMO guidelines
   b. NCCN guidelines
   c. CAP/IASLC/AMP guidelines
   d. ASCO guidelines
   e. USPSTF guidelines
   f. HHS/CMS guidelines
   g. Conferences
   h. Peers/Key Opinion Leaders (KOLs)
   i. Peer reviewed journals
   j. Tumor Board
   k. Independent Tech Assessments (e.g. UptoDate, ECRI, Hayes, MCG)
   l. Diagnostic test representatives
   m. Biopharma representatives
   n. Other (please specify) **[FREE TEXT]**

2. Which of the following do you consider to be the greatest challenges associated with traditional (non-digital) anatomic pathology today?

   *Please **rank your top 5** factors by dragging the factors into the box on the right, with the most influential reason at the top.*

   a. Peer consultation and/or consensus conference
   b. Preparing cases for tumor board
   c. Multi-hospital system requiring slide transport as part of workflow (time needed for transport and risk of slide damage/loss)
   d. Teaching trainees in groups
   e. Daily glass slide distribution
   f. Ergonomic issues with microscope us and/or desk space optimization of microscope/computer
   g. Requirement that pathologists be in office 5 days per week
   h. Desk space requirements for multi-headed scopes and computers
   i. Sharing microscopic pathology images with clinicians and/or in pathology reports
   j. Slide annotation
   k. Patient prior slide review
   l. Tissue stain diminished after prolonged storage
   m. Other (please specify) **[FREE TEXT]**

Please consider the following framework to define the different elements of digital pathology.

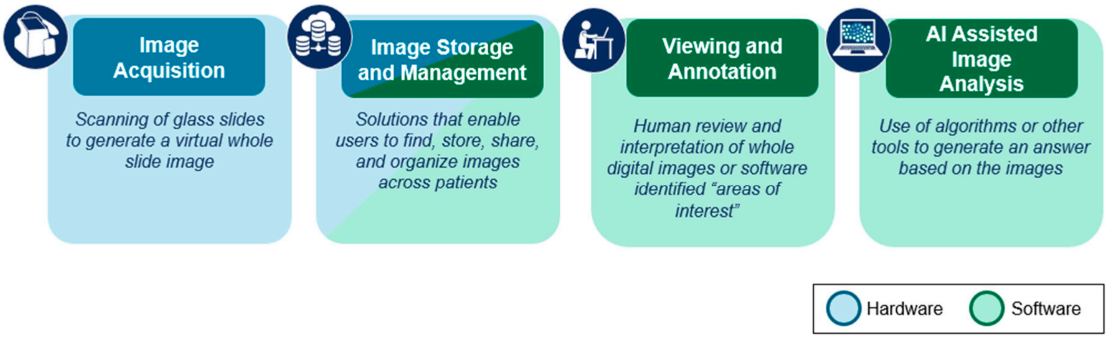

3. On a scale of 1 to 5, how well are each of the following challenges addressed by digital pathology, where 1 is not addressed and 5 is totally addressed?

Factor	1<br>Not Addressed	2	3<br>Somewhat Addressed	4	5<br>Totally Addressed
[ONLY DISPLAY OPTIONS IF SELECTED IN Q2]	[RADIO BUTTONS]				

4. On a scale of 1 to 5, please rate your level of enthusiasm for each element of digital pathology, where 1 is not enthusiastic and 5 is highly enthusiastic.

Please click the following link to review definitions of digital pathology elements.

Element	1 Not Enthusiastic	2	3 Somewhat Enthusiastic	4	5 Highly Enthusiastic
Image Acquisition	[RADIO BUTTONS]				
Image Storage and Management					
Image Viewing and Annotation					
AI guided image analysis					

5. On a scale of 1 to 5, which of the following value propositions of digital pathology are you most enthusiastic about, where 1 is not enthusiastic and 5 is highly enthusiastic?

Factor	1 Not Enthusiastic	2	3 Somewhat Enthusiastic	4	5 Highly Enthusiastic
Enable virtual/remote work model	[RADIO BUTTONS]				
Simplify and speed up external consultation					
Increase the volume of samples pathologist can review					
Streamline simple but laborious tasks (i.e., highlighting ROI for manual inspection), allowing pathologists to focus on more complex tasks					
Enable analysis of slides by non-specialist pathologists					
Allow for centralized analysis by subspecialists (e.g., GU pathologists)					
Improve accuracy and consistency in slide review with AI algorithm support					
Reduce the turnaround time (TAT) to definitive diagnosis					
Specific diagnosis/prognosis made possible only by AI algorithm					
Cost savings due to improved efficiency / productivity					
Cost savings due to reduction of slide storage needs					

Cost savings due to reduced pathologist staffing requirements	
Other (please specify) **[FREE TEXT]**	

6. Which of the following elements of digital pathology are required to realize each of the selected value propositions.

Please click the following link to review definitions of digital pathology elements.

Factor	Image acquisition	Image management and Storage	Image Viewing and Annotation	AI guided image analysis
**[ONLY SHOW OPTION IF SELECTED IN Q5 AS 4 OR 5]**	**[RADIO BUTTONS]**			

7. Which of the following elements of digital pathology has your lab adopted? *Please select all that apply.*

Please click the following link to review definitions of digital pathology elements.

a. Image acquisition
b. Image storage and management
c. Image viewing and annotation
d. AI guided image analysis
e. We have not adopted digital pathology yet **[EXCLUSIVE OPTION]**

8. **[IF Q7≠E]** For which of the following use cases are you using digital pathology today? *Please select all that apply.*

a. Remote interpretation (e.g., frozen section, cytology adequacy)

b. Primary diagnosis

c. Telepathology/ consultation (intra-, inter-institutional)

d. Sharing at tumor board

e. Cell counting (e.g., PD-L1, Ki67)

f. Region of interest highlighting

g. Annotating slides

h. Biomarker analysis using algorithms

i. Digital archiving

j. Research

k. Education and training

l. Other (please specify) **[FREE TEXT]**

9. **[IF Q7≠E]** Where is the majority of each element of digital pathology performed?

Please click the following link to review definitions of digital pathology elements.

	**[IF Q7=A] Image Acquisition**	**[IF Q7= B] Image Storage and Management**	**[IF Q7= C] Image Viewing and Annotation**	**[IF Q7=D] AI guided image analysis**
In my lab	**[RADIO BUTTONS]**			
In another lab within my hospital system				
Sent out to another reference or specialty lab				

10. **[IF Q7≠E AND Q9=IN MY LAB OR ANOTHER LAB IN HOSPITAL SYSTEM]** What types of FTEs are dedicated to digital pathology in your lab? *Please select all that apply.*

a. Retrained histopathology technologist

b. Retrained anatomic pathology laboratory technician

c. Retrained non-anatomic pathology laboratory technician9

d. Newly hired digital pathology technician

e. Other (please specify) **[FREE TEXT]**

11. **[IF Q7≠E AND Q9=IN MY LAB OR ANOTHER LAB IN HOSPITAL SYSTEM]** On a scale of 1 to 5, how challenging was it to hire, onboard, and train FTEs for digital pathology, where 1 is not challenging and 5 is very challenging?

Factor	1<br>Not Challenging	2	3<br>Somewhat Challenging	4	5<br>Very Challenging
Hiring, onboarding, and training FTEs for digital pathology	**[RADIO BUTTONS]**				

12. **[IF Q7=A]** Which of the following factors were most influential to digital pathology vendor selection for **image acquisition**?

*Please **rank your top 3** factors by dragging the factors into the box on the right, with the most influential reason at the top.*

Please click the following link to review definitions of digital pathology elements.

a. Quality and validation

b. Cost

c. Brand recognition

d. Customer support

e. Pre-existing relationships

f. Ease of use

g. Integration with workflow

h. EMR integration

i. One-stop shop

j. I had no oversight over vendor selection **[MAKE EXCLUSIVE]**

k. Other (please specify) **[FREE TEXT]**

13. **[IF Q7=A]** Which of the following best describes the **image acquisition** adoption process?
Please click the following link to review definitions of digital pathology elements.

a. Very challenging
b. Somewhat challenging
c. Neutral
d. Somewhat easy
e. Very easy

14. **[IF Q7=A]** What are the greatest challenges with **image acquisition**?

*Please **rank your top 3** factors by dragging the factors into the box on the right, with the most impactful reason at the top.*

Please click the following link to review definitions of digital pathology elements.

a. Lack of reimbursement
b. Budgetary constraints deprioritize the need to adopt high-cost DP solutions
c. Logistical challenge with training staff to incorporate DP
d. Reluctance from administrative stakeholders
e. Difficulty in setting up/ maintaining IT integration
f. Concerns/ challenges in maintaining data security
g. Lack of clear clinical evidence of the benefits of digital pathology adoption
h. Lack of peers' recommendations/referral on the digital pathology platform
i. Insufficient scale to make investment in image acquisition hardware cost-effective
j. Additional hands-on time required to load and scan slides
k. Poor customer service from vendors
l. Scanners require maintenance too frequently
m. High-resolution scans are too slow
n. Other (please specify) **[FREE TEXT]**

15. **[IF Q7=A]** Which of the following stakeholders were involved in the **image acquisition** adoption process? *Please select all that apply.*

Please click the following link to review definitions of digital pathology elements.

a. Lab director

b. Pathologists

c. Medical oncologists

d. Surgeons

e. Other providers

f. Hospital administrators

g. IT

h. Other (please specify) **[FREE TEXT]**

16. **[IF Q7=A]** On a scale of 1 to 5, how supportive was institutional leadership from each of the following stakeholder groups in the adoption of **image acquisition**, where 1 is not supportive and 5 is highly supportive.

Please click the following link to review definitions of digital pathology elements.

Stakeholder	1 Not Supportive	2	3 Somewhat Supportive	4	5 Highly Supportive
**[ONLY SHOW OPTION IF SELECTED IN Q15]**					

17. **[IF Q7=A AND Q16>2]** How influential were the following messages on convincing institutional leadership to adopt **image acquisition**?

Please ***rank your top 3*** *factors by dragging the factors into the box on the right, with the most influential reason at the top.*

Please click the following link to review definitions of digital pathology elements.

a. Enable virtual/remote work model

b. Simplify and speed up external consultation

c. Improve productivity with AI-algorithms

d. Increase the volume of samples pathologist can review

e. Streamline simple but laborious tasks (i.e., highlighting ROI for manual inspection), allowing pathologists to focus on more complex tasks

f. Enable analysis of slides by non-specialist pathologists

g. Allow for centralized analysis by subspecialists (e.g., GU pathologists)

h. Improve accuracy and consistency in slide review

i. Reduce the TAT to definitive diagnosis

j. Specific diagnosis/prognosis made possible only by AI algorithm

k. Cost savings due to improved efficiency / productivity

l. Cost savings due to reduction of slide storage needs

m. Cost savings due to reduced staffing requirements

n. Other (please specify) **[FREE TEXT]**

18. **[IF Q7=B]** Which of the following factors were most influential to digital pathology vendor selection for **image storage and management**?

Please ***rank your top 3*** *factors by dragging the factors into the box on the right, with the most influential reason at the top.*

Please click the following link to review definitions of digital pathology elements.

a. Quality and validation

b. Cost

c. Brand recognition

d. Customer support

e. Pre-existing relationships

f. Ease of use

g. Integration with workflow

h. EMR integration

i. One-stop shop

j. I had no oversight over vendor selection **[MAKE EXCLUSIVE]**

k. Other (please specify) **[FREE TEXT]**

19. **[IF Q7=B]** Which of the following best describes the **image storage and management** adoption process?

Please click the following link to review definitions of digital pathology elements.

a. Very challenging

b. Somewhat challenging

c. Neutral

d. Somewhat easy

e. Very easy

20. **[IF Q7=B]** What are the greatest challenges with **image storage and management**?

*Please **rank your top 3** factors by dragging the factors into the box on the right, with the most impactful reason at the top.*

Please click the following link to review definitions of digital pathology elements.

a. Lack of reimbursement

b. Budgetary constraints deprioritize the need to adopt high-cost DP solutions

c. Logistical challenge with training staff to incorporate DP

d. Reluctance from administrative stakeholders

e. Difficulty in setting up/ maintaining IT integration

f. Concerns/ challenges in maintaining data security

g. Paid solutions provide insufficient value-add over alternative file storage options

h. Lack of standardization of image file formats

i. Lack of interoperability among platforms

j. Insufficient scale to make investment in image storage hardware/software cost-effective

k. Poor customer service from vendors

l. Software updates from the vendor break LIS/EMR integration

m. Image files are too large to easily share

n. Unattractive financial terms for licensing software

o. Other (please specify) **[FREE TEXT]**

21. **[IF Q7=B]** Which of the following stakeholders were involved in the **image storage and management** adoption process? *Please select all that apply.*

Please click the following link to review definitions of digital pathology elements.

a. Lab director

b. Pathologist

c. Medical oncologists

d. Surgeons

e. Other providers

f. Hospital administrators

g. IT

h. Other (please specify) **[FREE TEXT]**

22. **[IF Q7=B]** On a scale of 1 to 5, how supportive was institutional leadership from each of the following stakeholder groups in the adoption of **image storage and management**, where 1 is not supportive and 5 is highly supportive.

Please click the following link to review definitions of digital pathology elements.

Stakeholder	1 Not Supportive	2	3 Somewhat Supportive	4	5 Highly Supportive
**[ONLY SHOW OPTION IF SELECTED IN Q21]**					

23. **[IF Q7=B AND Q22>2]** How influential were the following messages on convincing institutional leadership to adopt **image storage and management**?

Please ***rank your top 3*** *factors by dragging the factors into the box on the right, with the most influential reason at the top.*

Please click the following link to review definitions of digital pathology elements.

a. Enable virtual/remote work model

b. Simplify and speed up external consultation

c. Improve productivity with AI-algorithms

d. Increase the volume of samples pathologist can review

e. Streamline simple but laborious tasks (i.e., highlighting ROI for manual inspection), allowing pathologists to focus on more complex tasks

f. Enable analysis of slides by non-specialist pathologists

g. Allow for centralized analysis by subspecialists (e.g., GU pathologists)

h. Improve accuracy and consistency in slide review

i. Reduce the TAT to definitive diagnosis

j. Specific diagnosis/prognosis made possible only by AI algorithm

k. Cost savings due to improved efficiency / productivity

l. Cost savings due to reduction of slide storage needs

m. Cost savings due to reduced staffing requirements

n. Archived slide images are higher quality than archived tissue blocks

o. Other (please specify) **[FREE TEXT]**

24. **[IF Q7=C]** Which of the following factors were most influential to digital pathology vendor selection for **image viewing and annotation**?

*Please **rank your top 3** factors by dragging the factors into the box on the right, with the most influential reason at the top.*

Please click the following link to review definitions of digital pathology elements.

a. Quality and validation

b. Cost

c. Brand recognition

d. Customer support

e. Pre-existing relationships

f. Ease of use

g. Integration with workflow

h. EMR integration

i. One-stop shop

j. I had no oversight over vendor selection **[MAKE EXCLUSIVE]**

k. Other (please specify) **[FREE TEXT]**

25. **[IF Q7=C]** Which of the following best describes the **image viewing and annotation** adoption process?

Please click the following link to review definitions of digital pathology elements.

a. Very challenging

b. Somewhat challenging

c. Neutral

d. Somewhat easy

e. Very easy

26. **[IF Q7=C]** What are the greatest challenges with **image viewing and annotation**?

Please ***rank your top 3*** *factors by dragging the factors into the box on the right, with the most impactful reason at the top.*

Please click the following link to review definitions of digital pathology elements.

a. Difficulties with annotating ROI

b. Lack of reimbursement

c. Budgetary constraints deprioritize the need to adopt high-cost DP solutions

d. Logistical challenge with training staff to incorporate DP

e. Reluctance from administrative stakeholders

f. Difficulty in setting up/ maintaining IT integration

g. Concerns/ challenges in maintaining data security

h. Lack of file format standardization

i. Lack of interoperability among platforms

j. Insufficient scale to make investment in image viewing software cost-effective

k. Additional hands-on time required to load and scan slides

l. Poor customer service from vendors

m. Software updates from the vendor break LIS/EMR integration

n. Lack of standardization of file formats

o. Resistance from colleagues to analyzing digitized versus glass slides

p. Unattractive financial terms for licensing software

q. Other (please specify) **[FREE TEXT]**

27. **[IF Q7=C]** Which of the following stakeholders were involved in the **image viewing and annotation** adoption process? *Please select all that apply.*

Please click the following link to review definitions of digital pathology elements.

a. Lab director

b. Pathologist

c. Medical oncologists

d. Surgeons

e. Other providers

f. Hospital administrators

g. IT

h. Other (please specify) **[FREE TEXT]**

28. **[IF Q7=C]** On a scale of 1 to 5, how supportive was institutional leadership from each of the following stakeholder groups in the adoption of **image viewing and annotation**, where 1 is not supportive and 5 is highly supportive.

Please click the following link to review definitions of digital pathology elements.

Stakeholder	1 Not Supportive	2	3 Somewhat Supportive	4	5 Highly Supportive
**[ONLY SHOW OPTION IF SELECTED IN Q27]**					

29. **[IF Q7=C AND Q28>2]** How influential were the following messages on convincing institutional leadership to adopt **image viewing and annotation**?

*Please **rank your top 3** factors by dragging the factors into the box on the right, with the most influential reason at the top.*

Please click the following link to review definitions of digital pathology elements.

a. Enable virtual/remote work model

b. Simplify and speed up external consultation

c. Improve productivity with AI-algorithms

d. Increase the volume of samples pathologist can review

e. Streamline simple but laborious tasks (i.e., highlighting ROI for manual inspection), allowing pathologists to focus on more complex tasks

f. Enable analysis of slides by non-specialist pathologists

g. Allow for centralized analysis by subspecialists (e.g., GU pathologists)

h. Improve accuracy and consistency in slide review

i. Reduce the TAT to definitive diagnosis

j. Specific diagnosis/prognosis made possible only by AI algorithm

k. Cost savings due to improved efficiency / productivity

l. Cost savings due to reduction of slide storage needs

m. Cost savings due to reduced staffing requirements

n. Other (please specify) **[FREE TEXT]**

30. **[IF Q7=D]** Which of the following factors were most influential to vendor selection for **AI guided image analysis**?

*Please **rank your top 3** factors by dragging the factors into the box on the right, with the most influential reason at the top.*

Please click the following link to review definitions of digital pathology elements.

a. Quality and validation

b. Cost

c. Brand recognition

d. Customer support

e. Pre-existing relationships

f. Ease of use

g. Integration with workflow

h. EMR integration

i. One-stop shop

j. I had no oversight over vendor selection **[MAKE EXCLUSIVE]**

k. Other (please specify) **[FREE TEXT]**

31. **[IF Q7=D]** Which of the following best describes the **AI guided image analysis** adoption process?

Please click the following link to review definitions of digital pathology elements.

a. Very challenging

b. Somewhat challenging

c. Neutral

d. Somewhat easy

e. Very easy

32. **[IF Q7=D]** What are the greatest challenges with **AI guided image analysis**?

Please ***rank your top 3*** *factors by dragging the factors into the box on the right, with the most impactful reason at the top.*

Please click the following link to review definitions of digital pathology elements.

a. Difficulties sending the specimen out for analysis if not performed in house

b. Difficulties receiving and reporting results if not performed in house

c. Lack of reimbursement

d. Budgetary constraints deprioritize the need to adopt high-cost DP solutions

e. Logistical challenge with training staff to incorporate DP

f. Reluctance from administrative stakeholders

g. Difficulty in setting up/ maintaining IT integration

h. Concerns/ challenges in maintaining data security

i. Lack of clear clinical evidence of the benefits of digital pathology adoption

j. Lack of peers' recommendations/referral on the digital pathology platform

k. Lack of interoperability among platforms

l. Lack of concordance between algorithms

m. Lack of clinical guideline recommendation for automated analysis

n. Poor customer service from vendors

o. Software updates from the vendor break LIS/EMR integration

p. Lack of standardization of file formats

q. Resistance from colleagues to analyzing digitized versus glass slides

r. Unattractive financial terms for licensing software

s. Lack of transparency/explanation of how algorithms work

t. Technology is not yet sufficiently mature for routine clinical use

u. Other (please specify) **[FREE TEXT]**

33. **[IF Q7=D]** Which of the following stakeholders were involved in the **AI guided image analysis** adoption process? *Please select all that apply.*

Please click the following link to review definitions of digital pathology elements.

a. Lab director

b. Pathologist

c. Medical oncologists

d. Surgeons

e. Other providers

f. Hospital administrators

g. IT

h. Other (please specify) **[FREE TEXT]**

34. **[IF Q7=D]** On a scale of 1 to 5, how supportive was institutional leadership from each of the following stakeholder groups in the adoption of **AI guided image analysis**, where 1 is not supportive and 5 is highly supportive.

Please click the following link to review definitions of digital pathology elements.

Stakeholder	1 Not Supportive	2	3 Somewhat Supportive	4	5 Highly Supportive
**[ONLY SHOW OPTION IF SELECTED IN Q33]**					

35. **[IF Q7=D AND Q34>2]** How influential were the following messages on convincing institutional leadership to adopt **AI guided image analysis**?

*Please **rank your top 3** factors by dragging the factors into the box on the right, with the most influential reason at the top.*

Please click the following link to review definitions of digital pathology elements.

a. Enable virtual/remote work model

b. Simplify and speed up external consultation

c. Improve productivity with AI-algorithms

d. Increase the volume of samples pathologist can review

e. Streamline simple but laborious tasks (i.e., highlighting ROI for manual inspection), allowing pathologists to focus on more complex tasks

f. Enable analysis of slides by non-specialist pathologists

g. Allow for centralized analysis by subspecialists (e.g., GU pathologists)

h. Improve accuracy and consistency in slide review

i. Reduce the TAT to definitive diagnosis

j. Specific diagnosis/prognosis made possible only by AI algorithm

k. Cost savings due to improved efficiency / productivity

l. Cost savings due to reduction of slide storage needs

m. Cost savings due to reduced staffing requirements

n. Other (please specify) **[FREE TEXT]**

36. **[IF Q7=D]** For which of the following solid tumor biomarkers have you adopted AI guided image analysis tools?

a. HER2 scoring

b. PD-L1 scoring

c. Ki67 scoring

d. None of the above **[MAKE EXCLUSIVE]**

e. Other (please specify) **[FREE TEXT]**

37. **[IF Q7= D]** How did your lab validate the AI guided image analysis tool?

a. Published validation studies

b. Combination of published studies and internal validation

c. Internal validation

d. Other (please specify) **[FREE TEXT]**

38. **[IF Q7= D]** How long did it take for your lab to validate your AI guided image analysis tool?

a. **[0-1000] months**

39. **[IF Q7= D]** How many FTEs were required to validate your AI guided image analysis tool? *Please include yourself if you were involved in the validation process.*

a. **[0-1000] FTEs**

40. **[IF Q7≠A, B, C AND D]** Which of the following elements of digital pathology are you likely to adopt in the next 3-5 years?

Please click the following link to review definitions of digital pathology elements.

Element	[IF Q7≠A] Image Acquisition	[IF Q7≠ B] Image Storage	[IF Q7≠ C] Image Viewing and Annotation	[IF Q7≠D] AI guided image analysis
In-house				
In another lab in my system				
Send out	**[RADIO BUTTONS]**			
Will not adopt				

41. **[IF Q7≠A, B, C AND D]** How long before you have fully adopted the following elements of digital and computational pathology? *Please consider how long from when you start discussions at your institution to when you have full transitioned fully digital and computational pathology use. Please click the following link to review definitions of digital pathology elements.*

a. **[IF Q40≠WILL NOT ADOPT]** Image acquisition **[FREE TEXT] Years**

b. **[IF Q40≠WILL NOT ADOPT]** Image storage and management **[FREE TEXT] Years**

c. **[IF Q40≠WILL NOT ADOPT]** Viewing and annotation **[FREE TEXT] Years**

d. **[IF Q40≠WILL NOT ADOPT]** AI guided image analysis **[FREE TEXT] Years**

42. **[IF Q7≠A]** How influential were the following barriers to not yet adopting **image acquisition**?

*Please **rank your top 3** factors by dragging the factors into the box on the right, with the most influential reason at the top.*

Please click the following link to review definitions of digital pathology elements.

a. Lack of reimbursement

b. Budgetary constraints deprioritize the need to adopt high-cost DP solutions

c. Logistical challenge with training staff to incorporate DP

d. Reluctance from administrative stakeholders

e. Difficulty in setting up/ maintaining IT integration

f. Concerns/ challenges in maintaining data security

g. Lack of clear clinical evidence of the benefits of digital pathology adoption

h. Lack of peers' recommendations/referral on the digital pathology platform

i. Insufficient scale to make investment in image acquisition hardware cost-effective

j. Additional hands-on time required to load and scan slides

k. Poor customer service from vendors

l. Scanners require maintenance too frequently

m. High-resolution scans are too slow

n. Other (please specify) **[FREE TEXT]**

43. **[IF Q7≠A]** Has **image acquisition** been formally evaluated by your lab?

Please click the following link to review definitions of digital pathology elements.

a. Yes

b. No

44. **[IF Q7≠A]** Which of the following stakeholders were involved or would be involved in the **image acquisition** adoption discussion process? *Please select all that apply.*

Please click the following link to review definitions of digital pathology elements.

a. Lab director

b. Pathologist

c. Providers

d. Hospital administrators

e. IT

f. Other (please specify) **[FREE TEXT]**

45. **[IF Q7≠B]** How influential were the following barriers to not yet adopting **image storage and management**?

Please ***rank your top 3*** *factors by dragging the factors into the box on the right, with the most influential reason at the top.*

Please click the following link to review definitions of digital pathology elements.

a. Lack of reimbursement

b. Budgetary constraints deprioritize the need to adopt high-cost DP solutions

c. Logistical challenge with training staff to incorporate DP

d. Reluctance from administrative stakeholders

e. Difficulty in setting up/ maintaining IT integration

f. Concerns/ challenges in maintaining data security

g. Paid solutions provide insufficient value-add over alternative file storage options

h. Lack of standardization of image file formats

i. Lack of interoperability among platforms

j. Insufficient scale to make investment in image storage hardware/software cost-effective

k. Poor customer service from vendors

l. Software updates from the vendor break LIS/EMR integration

m. Image files are too large to easily share

n. Unattractive financial terms for licensing software

o. Other (please specify) **[FREE TEXT]**

46. **[IF Q7≠B]** Has **image storage and management** been formally evaluated by your lab?

Please click the following link to review definitions of digital pathology elements.

a. Yes

b. No

47. **[IF Q7≠B]** Which of the following stakeholders were involved or would be involved in the adoption discussion process for **image storage and management**? *Please select all that apply.*

Please click the following link to review definitions of digital pathology elements.

a. Lab director

b. Pathologist

c. Providers

d. Hospital administrators

e. IT

f. Other (please specify) **[FREE TEXT]**

48. **[IF Q7≠C]** How influential were the following barriers to not yet adopting **image viewing and annotation**?

Please ***rank your top 3*** *factors by dragging the factors into the box on the right, with the most influential reason at the top.*

Please click the following link to review definitions of digital pathology elements.

a. Difficulties with annotating ROI

b. Lack of reimbursement

c. Budgetary constraints deprioritize the need to adopt high-cost DP solutions

d. Logistical challenge with training staff to incorporate DP

e. Reluctance from administrative stakeholders

f. Difficulty in setting up/ maintaining IT integration

g. Concerns/ challenges in maintaining data security

h. Lack of file format standardization

i. Lack of interoperability among platforms

j. Insufficient scale to make investment in image viewing software cost-effective

k. Additional hands-on time required to load and scan slides

l. Poor customer service from vendors

m. Software updates from the vendor break LIS/EMR integration

n. Lack of standardization of file formats

o. Resistance from colleagues to analyzing digitized versus glass slides

p. Unattractive financial terms for licensing software

q. Other (please specify) **[FREE TEXT]**

49. **[IF Q7≠C]** Has **image viewing and annotation** been formally evaluated by your lab?

Please click the following link to review definitions of digital pathology elements.

a. Yes

b. No

50. **[IF Q7≠C]** Which of the following stakeholders were involved or would be involved in the adoption discussion process for **image viewing and annotation**? *Please select all that apply.*

Please click the following link to review definitions of digital pathology elements.

a. Lab director

b. Pathologist

c. Providers

d. Hospital administrators

e. IT

f. Other (please specify) **[FREE TEXT]**

51. **[IF Q7≠D]** How influential were the following barriers to not yet adopting **AI guided image analysis**?

*Please **rank your top 3** factors by dragging the factors into the box on the right, with the most influential reason at the top.*

Please click the following link to review definitions of digital pathology elements.

a. Difficulties sending the specimen out for analysis if not performed in house

b. Difficulties receiving and reporting results if not performed in house

c. Lack of reimbursement

d. Budgetary constraints deprioritize the need to adopt high-cost DP solutions

e. Logistical challenge with training staff to incorporate DP

f. Reluctance from administrative stakeholders

g. Difficulty in setting up/ maintaining IT integration

h. Concerns/ challenges in maintaining data security

i. Lack of clear clinical evidence of the benefits of digital pathology adoption

j. Lack of peers' recommendations/referral on the digital pathology platform

k. Lack of interoperability among platforms

l. Lack of concordance between algorithms

m. Lack of clinical guideline recommendation for automated analysis

n. Poor customer service from vendors

o. Software updates from the vendor break LIS/EMR integration

p. Lack of standardization of file formats

q. Resistance from colleagues to analyzing digitized versus glass slides

r. Unattractive financial terms for licensing software

s. Lack of transparency/explanation of how algorithms work

t. Technology is not yet sufficiently mature for routine clinical use

u. Other (please specify) **[FREE TEXT]**

52. **[IF Q7≠D]** Has **AI guided image analysis** been formally evaluated by your lab?

Please click the following link to review definitions of digital pathology elements.

a. Yes

b. No

53. **[IF Q7≠D]** Which of the following stakeholders were involved or would be involved in the adoption discussion process for **AI guided image analysis**? *Please select all that apply.*

Please click the following link to review definitions of digital pathology elements.

a. Lab director

b. Pathologist

c. Providers

d. Hospital administrators

e. IT

f. Other (please specify) **[FREE TEXT]**

54. How do you expect the use of digital pathology **at your institution** to change in the next 3-5 years?

	Much Less Use	Somewhat Less Use	No Change	Somewhat More Use	Much More Use
Image Acquisition	**[RADIO BUTTONS]**				
Image Storage and Management					
Image Viewing and Annotation					
AI guided image analysis					

55. What are the strongest drivers to increasing digital pathology in the future?

*Please **rank your top 3** factors by dragging the factors into the box on the right, with the most influential reason at the top.*

a. More economical options
b. Broad adoption of digital pathology in partnered anatomical pathology labs
c. Scanner instruments with higher throughput become available
d. A comprehensive digital pathology solution offered by a trusted vendor
e. Improved interoperability of the digital pathology products on the market
f. More digital pathology scanner instruments receive FDA clearance
g. More primary diagnosis algorithms receive FDA approval
h. Guideline inclusion
i. More evidence supporting the use of automated analysis algorithms
j. Continuing shortage of trained pathologists and technicians
k. Reduced skepticism among colleagues and administrators as DP becomes more common
l. Early adopter mentality at your hospital/system
m. Other (please specify) **[FREE TEXT]**

56. Which of the current barriers pose the greatest hurdle to expanded use of digital pathology in 3-5 years?

*Please **rank your top 3** factors by dragging the factors into the box on the right, with the most influential reason at the top.*

a. Lack of reimbursement

b. Budgetary constraints deprioritize the need to adopt high-cost DP solutions

c. Logistical challenge with training staff to incorporate DP

d. Reluctance from administrative stakeholders

e. Difficulty in setting up/ maintaining IT integration

f. Concerns/ challenges in maintaining data security

g. Lack of clear clinical evidence of the benefits of digital pathology adoption

h. Lack of peers' recommendations/referral on the digital pathology platform

i. Poor customer service from vendors

j. Software updates from the vendor break LIS/EMR integration

k. Resistance from colleagues to analyzing digitized versus glass slides

l. Unattractive financial terms for licensing software

m. Lack of transparency/explanation of how algorithms work

n. Technology is not yet sufficiently mature for routine clinical use

o. Additional hands-on time required to load and scan slides

p. Insufficient scale to make investment cost-effective

q. Benefits of digital pathology overstated by vendors

r. Other (please specify) **[FREE TEXT]**

57. For which of the following use cases do you anticipate using digital pathology in the future? *Please select all that apply.*

a. Remote interpretation (e.g., frozen section, cytology adequacy)

b. Primary diagnosis

c. Telepathology/ consultation (intra-, inter-institutional)

d. Sharing at tumor board

e. Cell counting (e.g., PD-L1, Ki67)

f. Region of interest highlighting

g. Annotating slides

h. Biomarker analysis using algorithms

i. Digital archiving

j. Research

k. Education and training

l. Other (please specify) **[FREE TEXT]**

58. On a scale of 1 to 5, please rate how influential **guideline body recommendations for digital pathology** would be for each of the following factors, where 1 is not influential and 5 is very influential.

Factors	1 Not Influential	2	3 Somewhat Influential	4	5 Very Influential
Achieving reimbursement for digital pathology reimbursement	**[RADIO BUTTONS]**				
Securing buy-in from institutional leadership					
Promoting broader digital pathology uptake					
Promoting expanded training for digital pathology					

59. Please rate your level of agreement with the following statement: “A consensus statement from guideline bodies would be influential for promoting broader adoption of digital pathology.”

a. Strongly agree

b. Somewhat agree

c. Neutral

d. Somewhat disagree

e. Strongly disagree

60. On a scale of 1 to 5, what is your level of familiarity with algorithms for the analysis of solid tumor specimens, where 1 is not familiar and 5 is very familiar.

Algorithm	1 Not Familiar	2	3 Somewhat Familiar	4	5 Very Familiar
HER2 Scoring (low vs high)	[RADIO BUTTONS]				
PD-L1 Scoring					
Ki67 Scoring					
TROP2 Scoring					
Mutation status prediction based on cell morphology (e.g., detection of *EGFR* mutations)					
Cancer primary diagnosis algorithms (e.g., Paige Prostate Detect)					

61. Which of the following do you perceive as the value proposition of **AI guided image analysis for solid tumor biomarkers**?

*Please **rank your top 3** factors by dragging the factors into the box on the right, with the most influential reason at the top.*

a. Accurately determine scoring
b. Reduced TAT for biomarker test results
c. Ability to perform biomarker testing in-house
d. Enable detection of biomarkers that are difficult or impossible to manually score (e.g., HER2 ultra-low)
e. Automate tedious aspects of anatomic pathology
f. Offer a competitive advantage for the adopting hospital/system
g. Make the lab more attractive as site for clinical trials
h. Contribute to ongoing biomedical research
i. Allow for interpretation of biomarker test results by non-specialists
j. Reduce the cost of tumor biomarker testing
k. Democratize high-quality testing beyond KOL labs
l. Other (please specify) **[FREE TEXT]**

62. On a scale of 1 to 5, how challenging would it be to provide algorithmic scoring on a solid tumor specimen if requested by a provider in the near future, where 1 is not challenging and 5 is very challenging.

Solid Tumor Biomarker	1 Not Challenging	2	3 Somewhat Challenging	4	5 Very Challenging
**[ONLY SHOW OPTION IF RATED ≥3 IN Q60]**	**[RADIO BUTTONS]**				

63. **[IF Q62=4 OR 5]** For what reasons do you think it would be challenging to run algorithmic based AI guided image analysis?

*Please **rank your top 3** factors by dragging the factors into the box on the right, with the most influential reason at the top.*

a. We have not adopted digital pathology in-house today and wouldn't know where to send testing
b. Lack of reimbursement
c. Challenges with receiving results from a send out lab
d. Budgetary constraints preventing bringing it in house
e. Lack of time or bandwidth to train staff
f. Pushback from administration
g. Lack of IT infrastructure
h. Data security concerns
i. Lack of clear evidence for automated algorithms
j. Technology is not yet mature enough for routine clinical use
k. Other (please specify) **[FREE TEXT]**

64. **[IF Q61=1 OR 2]** For what reasons do you think it would **not** be challenging to run algorithm based AI guided image analysis? *Please select all that apply.*

a. We already perform automated AI guided image analysis for routine clinical use
b. We already perform automated image analysis for research purposes/clinical trials
c. We have already assessed the feasibility of offering algorithmic analysis in house
d. We would rely on send-out reference labs for analysis
e. We have experience implementing digital pathology technologies in our lab
f. Other (please specify) **[FREE TEXT]**

65. Which of the following would be most impactful for reducing barriers to adoption of algorithm-based AI guided image analysis? *Please select all that apply.*

a. On-site education (e.g., diagnostic liaisons)

b. CME dinners

c. Conference presentations

d. List of send-out labs capable of performing this analysis

e. Unbranded education materials

f. Branded marketing materials

g. Printed or web-based reference guides

h. Webinars/webcasts

i. Online CME

j. Coding/billing reference materials

k. Guideline incorporation

l. Other (please specify) **[FREE TEXT]**

66. Optional: Please share potential solutions to overcome barriers to digital and computational pathology.

[FREE TEXT]

67. Optional: Please provide us with any additional feedback on the survey.

[FREE TEXT]
